# Eagle syndrome: A rare neuropathic disorder affecting head and neck

**DOI:** 10.1097/MD.0000000000038128

**Published:** 2024-05-10

**Authors:** Zhenq Xu, Ping Shi, Ping Zhang

**Affiliations:** a Department of Oral and Maxillofacial Surgery, Tianjin Stomatological Hospital, School of Medicine, Nankai University; b Tianjin Key Laboratory of Oral and Maxillofacial Function Reconstruction, Tianjin Stomatological Hospital, School of medicine, Nankai University, China; c Pain Centre for Oral-Maxillofacial and Head-Neck Region, Tianjin Stomatological Hospital, School of medicine, Nankai University, China; d Department of Otorhinolaryngology, Tianjin Stomatological Hospital, School of medicine, Nankai University, China.

**Keywords:** Eagle syndrome, elongated styloid process, ossified stylohyoid ligament, psychological distress, styloidectomy

## Abstract

To investigate the clinical characteristics of Eagle syndrome (ES) and evaluate psychological distress of the patients. Ten cases of ES were enrolled, clinical characteristics and management were analyzed. Psychological disorders of the patients were assessed by the test of self-rating anxiety scale (SAS) and self-rating depression scale (SDS). There were 8 females and 2 males varying from 34 to 56 years with a mean age of 44.86 ± 8.38 years. The main complaints included foreign body sensation of pharynx, odynophagia, vertigo with turning of the head-neck, pain of anterolateral neck, and toothache. Three cases were right-side affected, 6 cases were left-sided and 1 case was bilateral. Radiographic examination showed the elongated styloid process of bilateral in all cases, however, hypertrophy, improper inclination, abnormal angulation of styloid process and more complete calcification of stylohoid ligament of the complained side were observed compared to the opposite side. Eight cases suffered from anxiety and/or depression. A surgical intervention was carried out on 6 patients to resect the elongated styloid process, the symptoms and mental distress disappeared after the operation and no recurrence was found in their follow-ups. Meticulous interrogation of illness history, proper examination, and radiological studies may be valuable in diagnostic confirmation of ES. It is the hyperostosis, abnormal angulation of the styloid process rather than the simple elongation which is more likely to be attributed to the development of ES. Psychological disorders in ES patients were observed in our study and should be paid more attention in the future research.

## 1. Introduction

The styloid process (SP) is a long, bony projection arising from the inferior surface of the Petros part of the temporal bone, situated anterior to the stylomastoid foramen. The accepted normal length of SP is usually no more than 2.5 cm and a measurement of 2.5 cm or longer is considered to indicate elongation, some researchers regard 3.0 cm as the upper limit of normal.^[1]^

Watt Eagle first combined a pain syndrome with an abnormal stylohyoid complex in 1937 and then expanded on his initial descriptions subsequently, termed Eagle syndrome (ES). ES is a seldom clinical condition that is usually caused by an elongated styloid process (SP) and/or a calcified stylohyoid complex. It has a large variety of presentations that range from mild discomfort of pharynx (such as a sensation of itching and/or foreign body in the throat) to pain in the head-and-neck region (such as dysphagia, tooth pain, hemifacial pain, otalgia and pain that radiates to the upper extremities) and even vertigo with turning-head.^[2,3]^

ES is commonly poorly recognized and probably mistreated as atypical facial pain, pharyngitis, tonsillitis, migraine, temporomandibular disorder, trigeminal neuralgia, and some kinds of ischemic diseases because of its low incidence and multivariate but nonspecific clinical features.^[4,5]^

A retrospective study of 10 cases of ES was carried out in our article and an illustrative case was presented to demonstrate a better understanding of its clinical characteristics and management, the underlying pathogenesis was discussed on the basis of radiographic manifestations.

Chronic pain is 1 of the main causes of physical and psychological distress, in which anxiety and depression are the most prevalent psychological disorders. Recurrent symptoms of ES can affect the daily life and work of the patients which often results in their mental distress. However, psychological disorders in ES patients are rarely a concerned to date, this topic was mentioned in our article.

## 2. Materials and methods

The investigation study was approved by the Tianjin Stomatological Hospital Ethics Committee (PH2009-W-066), and informed consent written was obtained from the patient and/or his family.

### 2.1. Collection of cases

Ten patients diagnosed as ES by the faculty of our department were collected from September 2009 to October 2023. Characteristics of the patients including gender, visiting age, affected side, main complaint, duration of sufferance, treatment approach, and outcome of following-up were analyzed.

### 2.2. Radiographic presentations

All the patients were examined by panoramic X-ray and/or CT. The radiographic features of SP (including length, hyperostosis, inclination, angulation) and distinctions between complained and normal sides were observed.

### 2.3. Assessment of psychological status of the patients

Self-rating anxiety scale (SAS) and self-rating depression scale (SDS) were tested when the patients were admitted to our department to evaluate their psychological status. The SAS was used to evaluate the severity of anxiety and a standard score >50 indicates involving in anxiety.^[6]^ The SDS was used as a measure to assess the severity of depressive symptoms and a standard score >53 indicates affecting by depression.^[7]^

### 2.4. Case presentation

A 34-year-old male came to our clinic with a main complaint of itching, cough, foreign body sensation, and pain during swallowing in the left throat for 3 years. He was advised to the department of otolaryngology, a diagnosis of pharyngitis and/or tonsillitis was made and drugs of anti-inflammation were prescribed; however, no effect was observed. An aggravating experience of nausea in the morning and vertigo when moving the head and neck from 1 side to the other occurred during the last year. He was then referred to the Department of Neurology, a diagnosis of ischemic vertigo was given but no improvement was gained after medication.

Extraoral examination showed no facial asymmetry. The sensation of vertigo could be triggered when the patient moved his head and neck to the left side. The patient experienced extreme tenderness when touching the left posterior and inner side of the mandibular angle and a solid mass was palpable in the same region. On an intraoral examination, there was no palpable mass or tenderness in the left tonsillar fossa.

**Figure 1.**
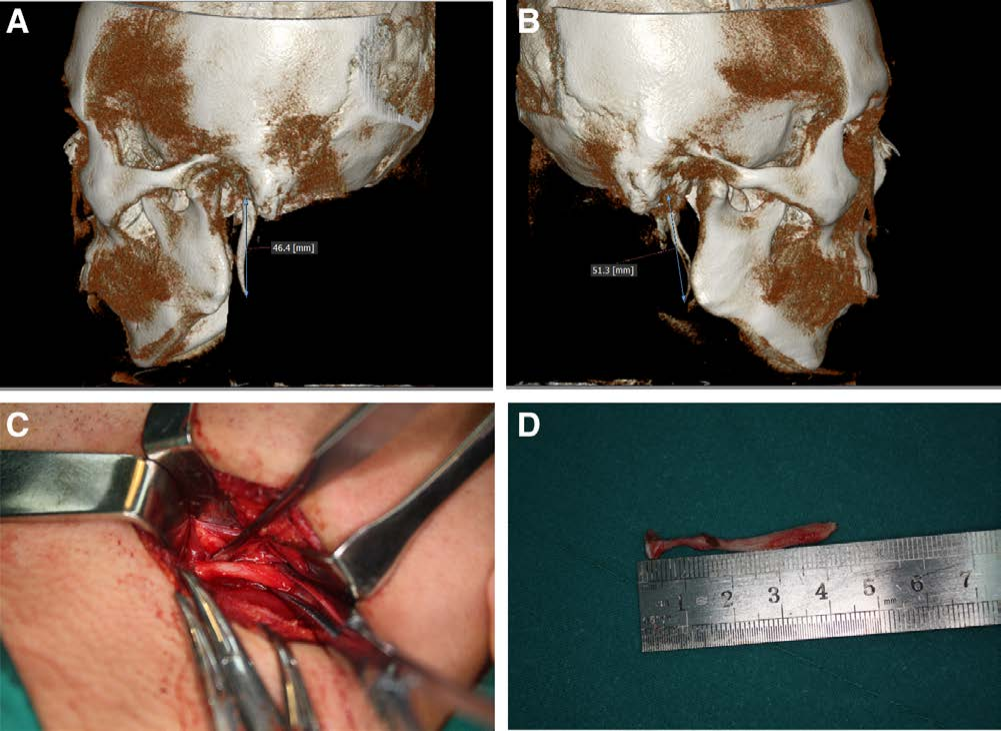
Illustrative presentation of Eagle syndrome (case 2). (A) CBCT features of the left styloid process. (B) CBCT features of the right styloid process. Bilateral elongated styloid processes were noted, however, the affected side of SP was more hypertrophy at the base and completely ossified at distal part as compared to the opposite side. (C) Exposition of the elongated styloid process through a cervical surgical approach. (D) Removal of the overlength of styloid process. A cervical surgical approach was performed and 4.1 cm length of the styloid process was removed. SP = styloid process.

An examination of CBCT revealed bilateral elongated styloid processes (left: 4.64 cm, right: 5.13 cm), of which the left process was more hypertrophy and completely ossified as compared to the right side (Fig. 1A and B).

Based on physical examination and radiographic analysis, the diagnosis of ES on left side and elongated SP on right side was made.

As the patient had failed medical management, surgical intervention was offered and the patient was keen on pursuing this option. After preparation, the elongated SP on the left side was removed through a cervical surgical approach under general anesthesia, and 4.1 cm length of the SP was resected (Fig. 1C and D). Since the patient had no complaints on the right side, the SP on that side was not excised.

Postoperative period was uneventful. The patient was free of symptoms the next day after the operation, and no recurrence was observed in 134-month follow-up.

## 3. Results

### 3.1. Characteristics of the cases

There were 8 females and 2 males in our research. The age of patients varied from 34 to 63 years with a mean age of 47.50 ± 9.11 years at the time of visit. Six cases were left-side involved, 3 cases were right-side affected and 1 case was both-side suffered. The main complaints included itching of throat, foreign body sensation of pharynx, cough, dysphagia, odynophagia, nausea, vertigo with turning of head-neck, and pain of anterolateral neck or tooth or face with a varying duration of 1 to 36 months (the mean suffering duration was 11.40 ± 10.08 months) (Table 1).

**Table 1:** Characteristics of cases.

Case	Sex	Age (yr)	Side affected	Complaint	Duration (mo)	Radiographic feature	Length (cm)		Psychological status	Treatment and approach
							R	L		
1	M	43	L	Odynophagia, sensation of foreign body	10	Elongated, tortuous angulation	5.10	4.58	Depression	Surgery and cervical approach
2	M	34	L	Dysphagia, sensation of foreign body, nausea, vertigo of head-neck turning	36	Elongated, improper inclination, hypertrophy	5.13	4.64	Anxiety, depression, suicide	Surgery and cervical approach
3	F	52	R	Anterolateral neck pain, nausea	18	Elongated, hypertrophy, calcified of ligament	5.89	6.68	Anxiety, depression	Surgery and cervical approach
4	F	56	R	Itching of throat, odynophagia, sensation of foreign body	15	Elongated, hypertrophy	4.91	5.92	Depression	Surgery and cervical approach
5	F	51	Bi	Pain of ear and neck	1	Elongated, hypertrophy	5.24	5.90	Normal	Surgery and cervical approach
6	F	42	L	Pain of throat, odynophagia	6	Elongated, hypertrophy	3.82	3.92	Anxiety, depression	Oral medication
7	F	36	L	Toothache	6	Elongated, hypertrophy	3.68	4.33	Anxiety	Surgery and oral approach
8	F	53	L	Pain of throat and ear, odynophagia	12	Elongated, hypertrophy	3.57	2.56	Anxiety, depression	Oral medication
9	F	63	R	Pain of throat and mandibular teeth	4	Elongated, hypertrophy	4.01	3.74	Normal	Oral medication
10	F	45	L	Pain of face and neck	6	Elongated, hypertrophy	3.48	3.55	Depression	Oral medication

Bi = bilateral, F = female, L = left, M = male, R = right.

### 3.2. Radiographic analysis

Radiographic examination showed the elongated SP of bilateral in all cases (right: 4.48 ± 0.86 cm; left: 4.58 ± 1.26 cm).

**Figure 2.**
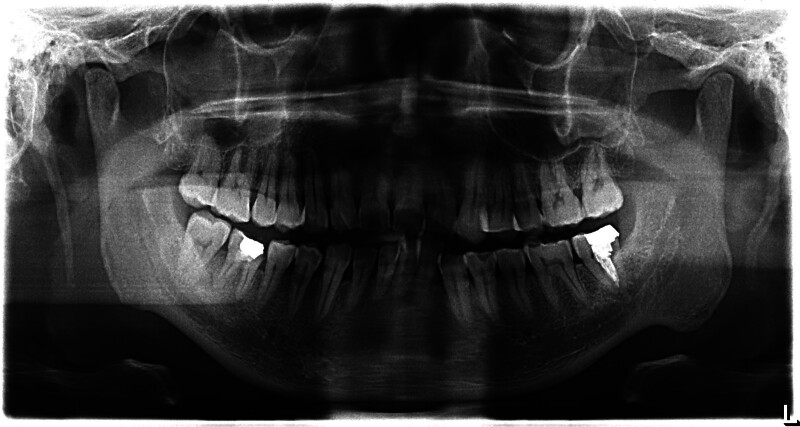
Panoramic feature of styloid process (case 1). The left styloid process manifested as more tortuous configuration and improper inclination versus the right.

The elongation SP of the complained side was unexpectedly shorter than the asymptomatic side in 5 cases. However, more hypertrophy, improper inclination, tortuous configuration of the SP, and completely ossification of stylohoid ligament in the affected side were observed compared to the opposite side in all the cases only 1 side complained (Figs. 1 and 2).

### 3.3. Psychological distress in cases

Two cases were normal, 1 case involved in anxiety, 2 cases suffered from depression, and 5 cases were affected by both anxiety and depression in our psychological survey. In the case of presentation, the sensation of a foreign body in the throat and vertigo disturbed his daily life and work so seriously that he even had a tendency of suicide (Table 1).

### 3.4. Management and follow-up of the cases

Oral anticonvulsants (carbamazepine, gabapentin) were administered to 4 cases because of their fear of surgery, however, only partial benefit was gained.

A surgical approach was carried out on 6 patients to resect the elongated SP (5 through cervical approach and 1 through oral approach), the symptoms including mental distress disappeared after intervention, and no recurrence was found with a mean following-up of 103.00 ± 53.23 months (Tables 1 and 2).

**Table 2:** Psychological distress improvement and follow-up of patients under styloid resection.

Case	SAS (standard score)		SDS (standard score)		Follow-up (mo)	Prognosis recurrence
	Preoperation	Postoperation	Preoperation	Postoperation		
1	49	36	66	51	169	No
2	65	40	64	50	134	No
3	59	40	61	48	127	No
4	46	43	56	50	97	No
5	49	41	50	49	74	No
7	55	45	55	48	17	No

SAS = self-rating anxiety scale, SDS = self-rating depression scale.

## 4. Discussion

The SP is the point of attachment for the bundle of Riolan, that is, the stylopharyngeus muscle, styloglossus muscle, and stylohyoid muscle as well as for the stylomandibular and stylohyoid ligaments. Numerous important anatomical neck structures are located in the direct vicinity of the SP, including IX to XII cranial nerves, internal carotid artery, external carotid artery, and internal jugular vein.^[1]^

Abnormality of SP (elongation, improper inclination, abnormal angulation) and/or an ossified stylohyoid ligament could compress and stretch the surrounding vessels and nerves, which lead to various symptoms, such as foreign body sensation, dysphagia, facial pain, presyncope, syncope, and even transient ischemic events.^[8]^ This condition was first described and discussed by Watt Eagle in 1937. He divided the syndrome into 2 forms: classic type and carotid artery type. The former includes symptoms such as foreign body sensation, pain referred to the neck, and dysphagia induced by impinging cranial nerves. The latter presents with other symptoms, such as migraines, neurological symptoms (such as parietal or periorbital pain), visual disturbances and syncope, caused by compression of the carotid artery and irritation of the sympathetic nerve plexus which coil the carotid arteries.^[2,3,9]^

ES is a rare pathological entity due to the low prevalence, vague, and diverse nature of presentation. The patients were usually recommended to the department of otorhinolaryngology, neurology, stomatology, pain clinic, even psychiatry and oncology, and misdiagnosed as head and neck inflammations (temporal arteritis, chronic tonsillitis, otitis media or externa, styloiditissubmandibular sialadenitis, pulpitis), cranial nerve disorders (trigeminal neuralgia, pterygopalatine ganglion neuralgia, glossopharyngeal neuralgia), temporomandibular joint disorders, presence of foreign bodies, and benign or malignant tumors within the head-neck region.^[1,4]^ All the cases were advised to be seen by many consultants like dentist, the nerve physician, neurosurgeon, the ear–nose–throat surgeon, and internist of pain specialist before they were clarified. In our study, 1 case (case 7) with her intolerable toothache was misdiagnosed as pulpitis and trigeminal neuralgia, root canal therapy was taken and gabapentin was used, however, hardly any improvement was gained, after resection the symptoms disappeared. Even 1 case (case 6) was admitted to see a psychiatrist and some antidepressants (lorazepam) were prescribed, only slight alleviation was benefited.

Previous epidemiological studies on ES reveal that it is more common in females above the age of 30 and right-side predominated, although the elongation of SP has been observed bilaterally, symptoms are usually experienced on 1 side only. The presenting symptoms include dull, aching pain on either side of the teeth, throat, difficulty in swallowing, foreign body sensation in the throat, pain in the facial region, and recurrent headache and vertigo.^[1,4]^ In our analysis, females were more involved equally to males with a ratio of 4:1 and with a mean visiting age of 47.50 ± 9.11 years. Radiographic examination showed elongated SP in both sides of all the patients, however, only 1 side was affected by ES in majority of the cases (9 in 10 cases, 3 in right and 6 in left). The main complaints included toothache, foreign body sensation of pharynx, odynophagia, syncope with turning of the head-neck, and pain of anterolateral neck.

The exact pathogenesis of ES remains unclear. There are many different etiologies that have been proposed to explain ES, such as congenital elongation due to persistence of cartilaginous precursors, posttraumatic scarring, and hyperplasia related to previous tonsillectomy. It is widely accepted that ES is associated with elongation of the SP and/or aberrant ossification of the stylohyoid apparatus. Thus, ES is also nominated and known as elongated SP or SP syndrome.^[1,4]^ The prevalence of SP elongation is reported to be 4%; however, only a small subgroup of these patients becomes symptomatic (between 4% and 10.3%).^[10,11]^ In few articles, some researchers pointed that some cases with normal length of SP can also performed ES.^[10]^ More interested in our article was that the length of SP in complaining side was shorter than that of the opposite side in some cases. While as comparison to the normal side, the SP thickened obviously, especially at the base, improper inclination and even torus angulation could be found. Stylohoid ligaments were ossified more completely in the affected side compared to the opposite side was also observed. Combination with our researches in radiographic tests, maybe it is the abnormalities in hypertrophy and aberrant inclination of SP or degree of calcification of stylohoid ligament rather than the simply extended length of SP which is more attributed to the occurrence of ES.

ES could be controlled by conservative methods of medical management, such as nonsteroidal anti-inflammatory drugs, anticonvulsants and antidepressants, however, this mode is usually inefficient.^[5,9]^ As shown in our research, little positive result was responded in cases from oral medication. The literature searched tend to support that surgical intervention should be the first choice which would result in more definitive treatment and long-lasting symptomatic relief. Surgical management is typically divided into the intraoral and cervical approaches.^[12,13]^ The elongated styloid processes of 5 patients in our research were palpated in the posterior of the mandibular angle, thus a cervical surgical approach was carried out to resect the elongated SP; for case 7, the SP was touched in the retro-molar region, an oral approach was taken. Symptoms of ES disappeared after operation and no recurrence was found with a mean following up of 103.00 ± 53.23 months which indicates that surgical removal of elongated SP is a permanent treatment protocol.

Chronic pain is usually accompanied by psychological disturbance, in which, anxiety and depression are the most commonly affected.^[14]^ Recurrent morbidity in ES often has an adverse impact on the state of well-being, precipitating the patient into psychological disorders. Two widely used self-report measures in the area of depression and anxiety are Zung self-rating depression scale (SDS) and self-rating anxiety scale (SAS). For the SDS and SAS, the cutoff points recommended by Zung^[15,16]^ were used: an index score of 45 and above (raw score 36 and above) for anxiety, an index score of 50 and above (raw score 40 and above) for depression. While for Chinese, the cutoff points for anxiety and depression are an index score of 50 and 53. In our study, 2 cases were free of mental illnesses attributing to their short duration and timely diagnosis. For the rest, they all suffered from anxiety and/or depression. Even for case 6, she was misdiagnosed as psychosis and oral antidepressants were recommended, however, she hardly benefited; she was willing to undergo surgery recently. For the case presented, the sensation of foreign body of the throat and vertigo with turning-head disturbed his daily life and work so seriously that he even had a tendency of suicide. The mental distress disappeared in the patients receiving operation in the following-up with the improvement of psychical pain.

## 5 Conclusions

The wide range of nonspecific clinical manifestations of ES may lead physicians to an inaccurate clinical diagnosis and gain the true diagnosis even more challenging. A thorough knowledge of the pain symptoms is very important, especially for the oral-maxillo-facial surgeon and ear–nose–throat surgeon. Persistent idiopathic facial pain (PIFP), previously named atypical facial pain is also 1 of the most challenging conditions to diagnose because of its variable symptoms. PIFP can affect any area of our face, especially jaw, ear or cheek area. The pain usually presents a long duration, lasting most or all of the day and is without sensory loss or other physical signs. Laboratory investigations including X-ray of face and jaws do not demonstrate relevant abnormality in PIFP patients.^[17,18]^ However, variation of SP can be observed in ES patients which may be a distinct difference between the 2 conditions. Anyone who is involved in head and neck pain without a specific etiology should be suspected as ES, and radiologic imaging especially CT should be added to make a confirmation. Surgical intervention can result in more definitive treatment and long-lasting symptomatic relief and should be accepted once ES is diagnosed. Psychological distress in patients affecting ES was observed in our study, such topic should be paid more attention in the future. An accurate diagnosis and timely intervention of ES is of great importance to relieve physical pain and ameliorate psychological diseases to help them regain normal daily life and work.

## Acknowledgment

Many thanks to Dr Yingb Yan and Jun Zhang for their support of our study.

## Author contributions

**Data curation:** Zhenq Xu, Ping Zhang.

**Investigation:** Zhenq Xu, Ping Shi.

**Methodology:** Zhenq Xu, Ping Zhang.

**Project administration:** Zhenq Xu.

**Writing – original draft:** Zhenq Xu.

**Writing – review & editing:** Zhenq Xu.
